# Supplementary material for: Immunogenicity of The BNT162b2 COVID-19 mRNA and ChAdOx1 nCoV-19 Vaccines in Patients with Hemoglobinopathies
Source: Vaccines (Basel). 2022 Jan 20;10(2):151. doi: 10.3390/vaccines10020151 (PMC8877446; doi:10.3390/vaccines10020151)
Supplement: Supplementary file 1 [file vaccines-10-00151-s001.zip › vaccines-1550866-supplementary.pdf]

Immunogenicity of The BNT162b2 COVID-19 mRNA and ChAdOx1 nCoV-19 Vaccines in Patients with Hemoglobinopathies

<u>Supplementary Document</u>

Table of contents

Table S1: COVID-19 binding antibodies correlation with the number of vaccine doses and the history of prior infection....2

Table S2: Characteristics of patients with transfusion-dependent thalassemia....3

Table S3: Characteristics of patients with sickle cell disease....4

Table S4: Comparison between time of sample collection and vaccine antibody response after first vaccine dose....5

Table S5: Comparison between time of sample collection and vaccine antibody response after second vaccine dose....6

Table S1: COVID-19 binding antibodies correlation with the number of vaccine doses and the history of prior infection

Characteristic	Category	Anti-S1 IgG		Anti-RBD IgG	
		positive	negative	positive	negative
No. of doses	One	32	0	32	0
	Two	34	0	34	0
History of COVID-19; Anti-N positive	Yes	31	0	31	0
	No	35	0	35	0

Table S2: Characteristics of patients with transfusion-dependent thalassemia

Characteristic	Category	Total n=51 (%)	Seropositive (n=43)	Seronegative (n=8)	*p*-Value
Gender[a]	Male	21 (41.1)	19 (44.2)	2 (25)	0.45
	Female	30 (58.9)	24 (55.8)	6 (75)	
Age[b]			31 (18-49)	32.5 (21-49)	0.65
No. of doses[a]	One	23 (45)	16 (37.2)	7 (87.5)	0.02
	Two	28 (55)	27 (62.8)	1 (12.5)	
Splenectomy[a]	Yes	30 (58.9)	24 (55.8)	6 (75)	0.45
	No	21 (41.1)	19 (44.2)	2 (25)	
Hydroxyurea[a]	Yes	4 (7.8)	4 (9.3)	0 (0)	1.0
	No	47 (92.2)	39 (90.7)	8 (100)	
Hyperferritinemia (>1000 ug/mL) [a]	Yes	44 (86.2)	36 (83.7)	8 (100)	0.58
	No	7 (13.8)	7 (16.3)	0 (0)	
History of COVID-19[a]; Anti-N positive	Yes	23 (45)	23 (53.5)	0 (0)	0.006
	No	28 (55)	20 (46.5)	8 (100)	

[a] n (column percentage); [b] Mean (range)

Table S3: Characteristics of patients with sickle cell disease

Characteristic	Category	Total n=15 (%)	Seropositive (n=13)	Seronegative (n=2)	*p*-Value
Gender[a]	Male	6 (40)	4 (30.8)	2 (100)	0.14
	Female	9 (60)	9 (69.2)	0 (0)	
Age[b]			31.2 (18-39)	38.5 (38-39)	0.92
No. of doses[a]	One	9 (60)	7 (53.9)	2 (100)	0.49
	Two	6 (40)	6 (92.3)	0 (0)	
Splenectomy[a]	Yes	1 (6.6)	1 (7.7)	0 (0)	1.0
	No	14 (93.4)	12 (44.2)	2 (100)	
Hydroxyurea[a]	Yes	13 (86.6)	11 (84.6)	2 (100)	1.0
	No	2 (13,4)	2 (15.4)	0 (0)	
Hyperferritinemia (>1000 ug/mL)[a]	Yes	10 (66.6)	9 (69.2)	1 (50)	1.0
	No	5 (33.3)	4 (30.8)	1 (50)	
History of COVID-19[a]; Anti-N positive	Yes	8 (53.3)	8 (61.5)	0 (0)	0.2
	No	7 (46.7)	5 (38.5)	2 (100)	

[a] n (column percentage); [b] Mean (range)

Table S4: Comparison between time of sample collection and vaccine antibody response after first vaccine dose

Time to sample collection	Less than three months n=37		Three months or more n=9		*p*-Value
	Median	Range	Median	Range	
nAbs	320	10-10240	80	10-5120	0.31
Anti-RBD	204800	1600-409600	25600	6400-409600	0.08
Anti-S1	204800	1600-409600	51200	6400-409600	0.14
Days from vaccination to collection	36	15-83	106	94-136	N/A

All the above tests were done using the Mann–Whitney test

Table S5: Comparison between time of sample collection and vaccine antibody response after second vaccine dose

Time to sample collection	Less than three months n=25		Three months or more n=9		*p*-Value
	Median	Range	Median	Range	
nAbs	5120	10-20480	1280	20-10240	0.1
Anti-RBD	409600	12800-409600	102400	12800-409600	0.24
Anti-S1	409600	12800-409600	204800	6400-409600	0.16
Days from vaccination to collection	50	17-88	106	93-155	N/A

All the above tests were done using the Mann–Whitney test
